# Osteosarcomatous Transformation in the Setting of Mazabraud's Syndrome: A Case Report and Review of the Literature

**DOI:** 10.1155/2019/2638478

**Published:** 2019-05-28

**Authors:** Iqbal Multani, Snezana Popovic, Naveen Parasu, Michelle Ghert

**Affiliations:** ^1^Royal North Shore Hospital, Reserve Rd, Sydney, New South Wales, Australia; ^2^Hamilton Health Sciences, Juravinski Hospital and Cancer Centre, 711 Concession Street, Hamilton, ON, Canada L8V 1C3; ^3^Centre for Evidence-Based Orthopaedics, Department of Surgery, McMaster University, 293 Wellington Street North, Suite 110, Hamilton, ON, Canada L8L 8E7

## Abstract

**Introduction:**

Mazabraud's Syndrome is a rare condition that is defined by the presence of fibrous dysplasia lesions in the bone and intramuscular myxomas in the soft tissue. Malignant transformation, in the setting of Mazabraud's Syndrome, of the fibrous dysplasia lesions into a sarcomatous neoplasm occurs in less than 1% of cases—with only six previously reported cases.

**Case Presentation:**

Here, we present a 62-year-old Caucasian female who developed an osteosarcoma in a fibrous dysplasia lesion of the proximal femur in the background of Mazabraud's Syndrome. The patient was treated with wide excision and endoprosthetic reconstruction. She declined adjuvant chemotherapy. She is alive without evidence of disease one-year postoperatively.

**Conclusion:**

Patients with Mazabraud's Syndrome remain at low risk for malignant transformation. However, close monitoring of asymptomatic patients with this condition for radiographic changes in their lesions and/or clinical symptoms is recommended.

## 1. Introduction

Mazabraud's Syndrome, named after the French physician Mazabraud in 1967 although first described by Henschen in 1926, is a rare disorder characterized by the presence of intramuscular myxoma(s) in association with monostotic or polyostotic fibrous dysplasia [1, 2]. With more than 100 reported cases of Mazabraud's Syndrome, there are only six published cases of sarcomatous transformation of the fibrous dysplasia lesions in this specific setting [1–8]. Here, we report the development of an osteosarcoma arising from a fibrous dysplasia lesion in the femur of a 62-year-old female in the background of Mazabraud's Syndrome.

## 2. Case Presentation

**Figure 1:**
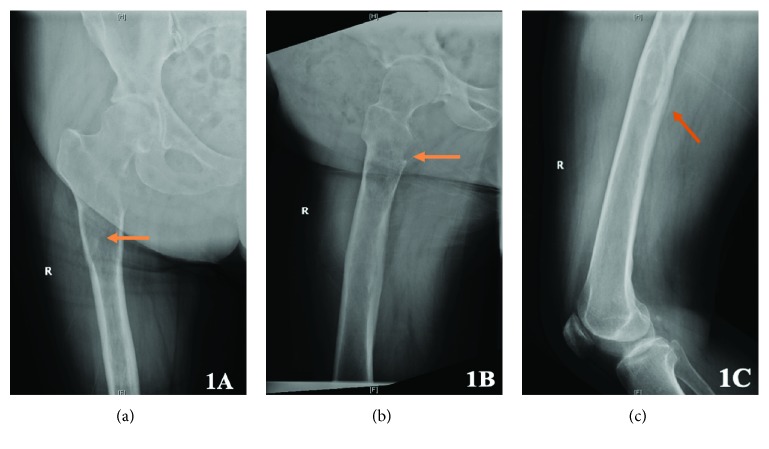
Initial anteroposterior and lateral radiographs of the right femur. (a) An expansile intramedullary lucent lesion is seen within the subtrochanteric region with no evidence of chondroid calcification. (b) Cortical destruction and lytic expansion of the lesser trochanter. (c) Ground-glass appearance of the mid femoral shaft with a sclerotic rim compatible with fibrous dysplasia.

A 62-year-old otherwise healthy woman presented with three to four months of mechanical right groin pain radiating to the right buttock. She had a history of a benign soft-tissue mass in the right thigh that had been biopsied 10 years earlier, and the patient was told it was a myxoma. No other workup was done at the time, and no other lesions were clinically detectable. She had been very active and played tennis regularly. She was assessed with a hip ultrasound for her right groin pain and was prescribed physiotherapy. During physiotherapy, she felt a snap in her right groin and was no longer able to ambulate without a walker. Initial radiographs demonstrated a lucent intramedullary lesion within the subtrochanteric region—further characterized by a focally aggressive appearance with cortical destruction and lytic expansion of the lesser trochanter (Figures 1(a) and 1(b)). A ground-glass appearance, typical of fibrous dysplasia, was also noted in the mid-femoral shaft (Figure 1(c)).

**Figure 2:**
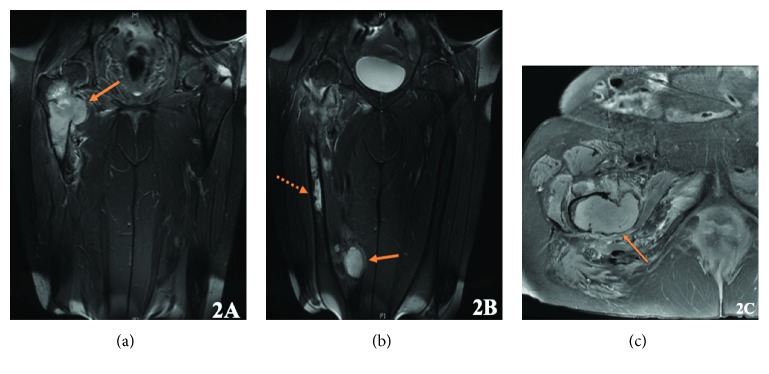
Fat-suppressed T2 MRI of the right femur. (a) Cortical destruction with an extraosseous soft-tissue mass in the region of the lesser trochanter. (b) Intermediate to high signal lesion within the medullary cavity of the proximal and mid right femoral shaft though indolent at the inferior aspect (dashed arrow). T2 hyperintense mass in the distal thigh in line with a myxoma (solid arrow).

Further workup included a Computed Tomography (CT) scan of the chest, abdomen, and pelvis which showed multiple hepatic and renal cysts but no evidence of a primary carcinoma or lung metastases. Bloodwork including serum protein electrophoresis was normal. A Total Body Bone Scan (TBBS) revealed increased uptake in the right proximal femur and two areas of relatively mild uptake in the mid femur. Fat-suppressed T2 Magnetic Resonance Imaging (MRI) displayed an intermediate to high signal lesion within the medullary cavity of the proximal and mid right femoral shaft (Figure 2(a)). In line with the initial radiographs in which two benign sites of fibrous dysplasia were noted, in the third proximal lesion, there was cortical destruction with an extraosseous soft-tissue mass in the region of the lesser trochanter. Additionally, in keeping with the known myxoma, a soft-tissue T2 hyperintense mass within the medial distal thigh was also present (Figure 2(b)). On further review of the MRI images, five other myxomas were seen. The imaging features were consistent with Mazabraud's Syndrome, with sarcomatous transformation of benign fibrous dysplasia in the proximal femur.

**Figure 3:**
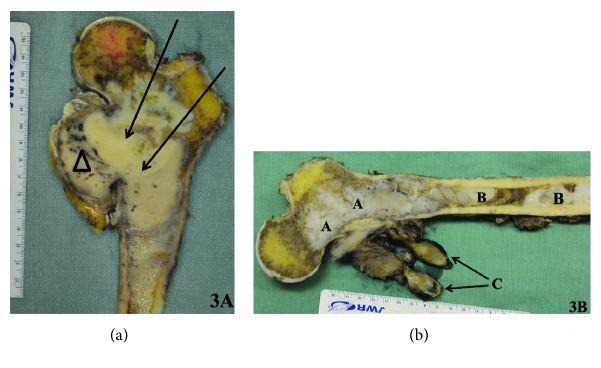
Gross pathology of the right femur. (a) Cut section of the femur showing an infiltrative, hard, tan-white tumor within the medullary cavity (arrows) invading the medial cortex and extending to soft tissue (triangle). (b) An infiltrative, hard, tan-white tumor (A), well-distinct white areas (B), and well-circumscribed soft-tissue myxoid lesion (C) corresponding microscopically to an osteosarcoma (A), fibrous dysplasia (B), and myxoma (C).

**Figure 4:**
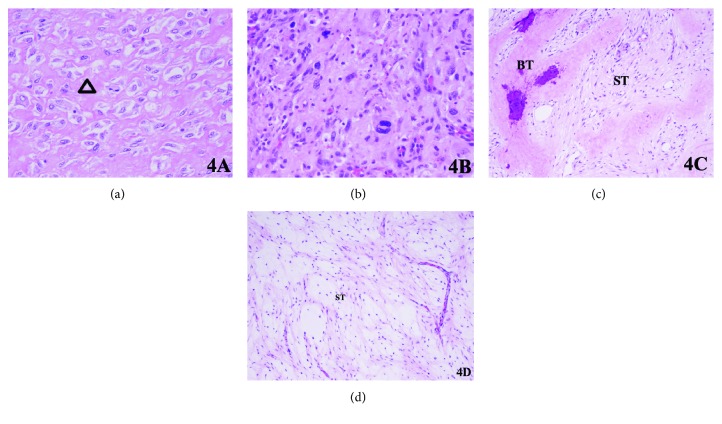
Microscopic hematoxylin and eosin stain sections of right femur tissue. (a) Osteosarcoma with osteoblastic component including neoplastic osteoid formation (triangle) and (b) markedly pleomorphic tumor cells. (c) Fibrous dysplasia with irregular bony spicules and trabeculae (BT) with fibrous stroma (ST). (d) Myxoma composed of hypocellular and hypovascular stromata (ST) containing abundant myxoid matrix and bland spindled cells.

Due to the high risk of fracture, the patient was immediately placed on bedrest and a biopsy was not performed—as a preventative measure to reduce fracture risk. She was taken to the operating room and underwent wide resection of the proximal right femur with endoprosthetic reconstruction via a lateral approach. Along with bony specimens, one of the soft-tissue masses was also excised and sent to pathology for investigation (Figures 3(a) and 3(b)). Pathology later confirmed a high-grade osteosarcoma in the setting of fibrous dysplasia and a benign myxoma (Figures 4(a)–4(d)). There were no complications during the procedure.

Postoperatively, the patient stayed in the hospital for one week, with physiotherapy beginning on postoperative day one. Her postoperative course followed the normal trajectory of recovery with no major complaints or issues at her two-week follow-up. The patient declined adjuvant chemotherapy. The standard management of osteosarcoma at our center would include wide surgical excision with neoadjuvant or adjuvant chemotherapy (methotrexate- and doxorubicin-based multidrug regimens). The patient is being followed to assess for complications and local or systemic relapse every three months with radiographs of the femur and chest. At one year postoperatively, there have been no complications or evidence of disease relapse.

## 3. Discussion

Fibrous dysplasia is typically a benign intramedullary fibroosseous lesion that is characterized by a disarrayed trabecula of woven bone separated by fibroconnective tissue [6, 7]. These lesions classically impact a single bone, with the femur being commonly involved. Malignant transformation of fibrous dysplasia is a rare event, with the most frequent sites of fibrous dysplasia and thus transformation being the craniofacial bones and proximal femur with secondary tumors inclusive of osteosarcoma, chondrosarcoma, and high-grade spindle cell sarcoma [7]. Myxomas are benign soft-tissue tumors defined by bland spindle- and stellate-shaped cells with a hypovascular, myxoid stroma [7]. To our knowledge, there have been no reported cases of malignant transformation of myxomas, although benign reoccurrences have been reported [7].

The association between fibrous dysplasia and intramuscular myxomas was first noted by Henschen in 1926, but the actual syndrome was self-named after Mazabraud in 1967 [1]. The general consensus of the recent literature is that both components of the syndrome are caused by the same postzygotic substitution mutation of *GNAS1*—a gene that codes for an alpha subunit for a stimulatory G-protein involved with cell proliferation [4, 7]. The McCune-Albright Syndrome also shares fibrous dysplastic lesion due to a similar postzygotic mutation though likely not as penetrative into soft tissues as indicated by the lack of myxomas [2, 7].

As in the current case, Mazabraud's Syndrome typically manifests as a polyostotic fibrous dysplasia with multiple intramuscular myxomas appearing close by [7]. The literature is indicative that the syndrome has a predisposition for females and often involves the long bones of the lower limbs bilaterally [4]. It is important to consider several differentials when approaching this presentation inclusive of the following: a primary bone malignancy, metastatic disease, multiple myeloma, lymphoma, and Paget's disease. Radiographic findings of the fibrous dysplasia lesions classically include cortical thickening, cystic translucencies, and a ground-glass appearance [1]. Generally, these lesions prelude the appearance of the intramuscular myxomas [9]. Management of Mazabraud's Syndrome is normally conservative unless there is functional impact.

With seven published cases, inclusive of the current case, the propensity for malignant transformation of a fibrous dysplastic lesion into a sarcomatous neoplasm in the setting of Mazabraud's Syndrome remains a rare event (Table 1). A brief review of the reported cases notes that the average age of diagnosis of a sarcoma is 53 years of age, with a near equal predilection for gender. Patients usually present with an insidious onset of pain. Osteosarcoma was the most common malignant diagnosis followed by spindle cell sarcoma and chondrosarcoma. Transformations occurred in the femur (3 cases), tibia (2 cases), humerus (1 case), and radius (1 case). All previously reported cases underwent biopsy prior to definitive treatment. Unique to our case, we relied on a preliminary diagnosis based on imaging studies rather than biopsy to initiate management so as to reduce the risk of pathologic fracture.

## 4. Conclusion

In summary, we present a case of an otherwise healthy 62-year-old female, with a known history of an untreated myxoma, diagnosed with a high-grade osteosarcoma secondary to Mazabraud's Syndrome. With only six previously published cases, this malignant transformation of an otherwise relatively benign syndrome clearly adds further weight to the previously reported cases. Although malignant transformation remains a rare event, close monitoring of asymptomatic patients with this condition for radiographic changes in their lesions and/or clinical symptoms is recommended.

## Figures and Tables

**Table 1:** Published studies of sarcomatous transformation of fibrous dysplasia in the setting of Mazabraud's Syndrome specifically (McCune-Albright studies are excluded).

Case	Author	Year	Title	Patient age	Gender	Diagnosis	Location	Presenting symptom
1	Roze, R. et al.	1967	Fibrous dysplasia of bone and myxomas of the soft tissues. Localized sarcomatous degeneration [French]	72	Female	Osteosarcoma	Right tibia (distal)	Pain
2	Witkin, G. et al.	1986	Osteogenic sarcoma and soft tissue myxoma in a patient with fibrous dysplasia and hemoglobins	41	Male	Osteosarcoma	Left tibia	Asymptomatic
3	Nyguen, B. et al.	2005	Mazabraud's Syndrome with sarcomatous transformation: scintigraphic and radiologic imaging	48	Male	Spindle cell sarcoma	Right humerus (proximal)	Pain
4	Crawford, E. et al.	2009	Osteosarcoma of the proximal part of the radius in Mazabraud syndrome. A case report	63	Male	Osteosarcoma	Right radius (proximal)	Pain
5	Szymanski, C. et al.	2015	Chondrosarcoma of the femur in Mazabraud's syndrome: a first case study	51	Female	Chondrosarcoma	Left femur	Pain and swelling
6	Calleja, M. et al.	2018	Mutational analysis of high-grade spindle cell sarcoma of the femur in Mazabraud's syndrome	36	Female	Spindle cell sarcoma	Right femur	Pain
7	Ghert, M. et al.	2018	Osteosarcomatous transformation in the setting of Mazabraud's Syndrome: a case report and review of the literature	62	Female	Osteosarcoma	Right femur (proximal)	Pain
